# Modelling population dynamics in a unicellular social organism community using a minimal model and evolutionary game theory

**DOI:** 10.1098/rsob.200206

**Published:** 2020-11-04

**Authors:** Ravindra Garde, Jan Ewald, Ákos T. Kovács, Stefan Schuster

**Affiliations:** 1 Department of Bioinformatics, Matthias Schleiden Institute, Friedrich Schiller University Jena, Ernst-Abbe-Platz 2, 07743 Jena, Germany; 2 Max Planck Institute for Chemical Ecology, Hans-Knöll-Strasse 8, 07745 Jena, Germany; 3 Bacterial Interactions and Evolution Group, DTU Bioengineering, Technical University of Denmark, Søltofts Plads Building 221, 2800 Kongens Lyngby, Denmark

**Keywords:** population dynamics, sporulation, biofilms, fruiting bodies, bet hedging, evolutionary game theory

## Abstract

Most unicellular organisms live in communities and express different phenotypes. Many efforts have been made to study the population dynamics of such complex communities of cells, coexisting as well-coordinated units. Minimal models based on ordinary differential equations are powerful tools that can help us understand complex phenomena. They represent an appropriate compromise between complexity and tractability; they allow a profound and comprehensive analysis, which is still easy to understand. Evolutionary game theory is another powerful tool that can help us understand the costs and benefits of the decision a particular cell of a unicellular social organism takes when faced with the challenges of the biotic and abiotic environment. This work is a binocular view at the population dynamics of such a community through the objectives of minimal modelling and evolutionary game theory. We test the behaviour of the community of a unicellular social organism at three levels of antibiotic stress. Even in the absence of the antibiotic, spikes in the fraction of resistant cells can be observed indicating the importance of bet hedging. At moderate level of antibiotic stress, we witness cyclic dynamics reminiscent of the renowned rock–paper–scissors game. At a very high level, the resistant type of strategy is the most favourable.

## 1. Introduction

Many unicellular organisms have been shown to exhibit social interactions. For instance, biofilms such as those of *Bacillus subtilis* [1], *Escherichia coli* [2], *Pseudomonas aeruginosa* and many other bacteria show distinct social dynamics including cooperation [3,4], competition [5,6], division of labour [7], altruism [8], cheating [2,9] and even cannibalism [10]. Such communities do not only represent an aggregation of cells but have long been observed as possible first steps towards the evolution of multicellularity [11]. Different subpopulations within the biofilm are subjected to different microenvironments [12]. Such microenvironments are a consequence of gradients of different substances like nutrients and waste products in the biofilm [13]. This often implies that different subpopulations exhibit distinct phenotypes. For example, a subpopulation of metabolically active cells in a *P. aeruginosa* biofilm develops resistance to colistin, whereas the metabolically inactive cells can survive ciprofloxacin and tetracycline. In order to destroy the biofilm, a treatment of colistin in combination with either ciprofloxacin or tetracycline is required [14]. Similarly, *B. subtilis* biofilm shows phenotypic differences in the cells located in the interior from those located in the periphery [15]. This system has been studied in detail by Liu *et al.* [15] and has been modelled using minimal models [16,17]. In this study, we aim to apply a similar minimal modelling approach in the context of population dynamics in a typical community of unicellular social organisms.

Fruiting bodies are observed in eukaryotes like *Dictyostelium discoideum* [18,19] and other dictyostelids. The amoebae *Acanthamoeba pyriformis* and *Luapelamoeba arachisporum* form minute sporocarpic fruiting bodies [20]. Moreover, prokaryotes including *Myxococcus xanthus* and *B. subtilis* among others have been proposed to form similar fruiting bodies [21,22]. Motile cells attach to one another and form aerial structures that are the hub for sporulation. These cells lose their motility in order to form the sporulating fruiting body. Our study explores what makes a particular cell take such a decision, to change its phenotype. To do this, we employ a minimal model consisting of three ordinary differential equations (ODEs) as originally proposed by Wilhelm & Heinrich [23] to describe oscillatory chemical reactions. This model has also been used to describe the periodic halting in the expansion of a *B. subtilis* biofilm. Furthermore, it has been used to study oscillations in the p53 system of higher eukaryotes [24].

**Figure 1.**
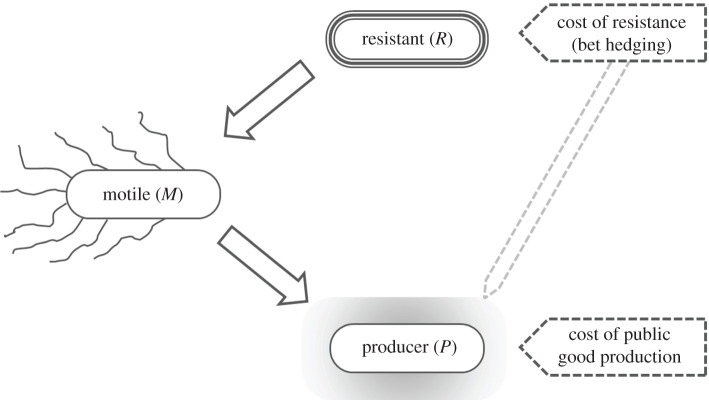
Scheme of the population dynamics of the three subpopulations of the model. *M* cells arise from *R* cells and can differentiate into *P* cells. The *P* cells incur costs of public goods production (indicated dashed pentagon) while the *R* cells are mainly responsible for bet hedging (indicated with dashed pentagon). *P* cells also affect the degree of bet hedging, for example, by contributing to fruiting body establishment.

Here, we focus on creating a generalized model that can be applied to a wide range of organisms that coexist in a community, in addition to relating the observations to *B. subtilis*. In that regard, the biofilm can be considered a public good, and the spores can be considered a highly resistant and persistent phenotype. We consider three subpopulations in the biofilm that exhibit three different phenotypes (figure 1).

— The resistant cells, which are the most resilient to nutrient scarcity and chemical attack.
— The producer cells, which can produce the public goods which help facilitate growth, but are also more susceptible to antibiotics and other chemical and environmental challenges.
— The motile cells, which have the ability to move away from antibiotics and towards nutrients (chemotaxis).

Each of these types is represented by an ODE. We were interested in the proportion of the resistant cells with respect to the producers; therefore, the population distribution for three different cases were studied each corresponding to a different range of antibiotic stress: when there is no antibiotic, moderate level of antibiotic and very high level of antibiotic.

We then describe the observations in each of the three cases using evolutionary game theory. We do this by considering a two-strategy game and a three-strategy game. Our study suggests that as the antibiotic stress increases, the system tends to stabilize towards a higher proportion of resistant cells. This state is reached through oscillations in the proportion of producers to resistant cells.

## 2. Methods

### 2.1. The model

We consider three subpopulations of an organism, as outlined above. The resistant subpopulation, denoted by *R*, which can survive antibiotics and environmental stresses (figure 1). This subpopulation can transit into a motile cell subpopulation denoted by *M*, which is a representative of free-floating cells with chemotaxis. It can also be regarded as an intermediate stage between the resistant phenotype changing into the producer phenotype, denoted by *P*. Subpopulation *P* pays the costs associated with the production of public goods.

Thus, the dynamics of these subpopulations can be described using three variables based on the model suggested by Wilhelm & Heinrich [23], as follows:

2.1*a* $$\frac{\text{d}R}{\text{d}t}=k_{1}NR-\;k_{4}R-k_{2}RP,$$

2.1*b* $$\frac{\text{d}P}{\text{d}t}=-k_{3}P\;+\;k_{5}M$$

2.1*c* $$\text{and}\;\frac{\text{d}M}{\text{d}t}=k_{4}R\;-\;k_{5}M.$$

Assumptions of the model:

— The subpopulations are generated in the order *R* —> *M* —> *P*.
— *R* cells show an arrested state of cell growth but show a self-amplifying proliferation because it serves the reproduction of the population. Such behaviour of *R* cells was also shown in *Dictyostelium* [18,19]*.* They can be thought of as a reserve of cells that have highly reduced functions and only exhibit resistance phenotypes. In a community such as a biofilm which show efficient nutrient sharing [25], *R* cells which have a highly restricted metabolism, and, therefore, very limited nutrient requirements, obtain a steady supply of nutrients quite easily. This is given by the constant *N*.
— The term *k*_2_*RP* represents that both subpopulations *R* and *P* are the essential components of the ‘sessile’ community. Without subpopulation *R*, the community will not be able to survive the biotic and abiotic stress and without subpopulation *P*, the public goods, the very basis for a communal lifestyle, would not be produced. For example, in a fruiting body, the spores (analogous to resistant cells in our model) and the biofilm (analogous to public goods in our model) matrix are both required in order to form a fruiting body. The term also represents the degree of bet hedging or ‘resistance’ the community invests in. It follows that a community that produces more public goods also invests more in bet hedging.
— The cost of public goods production is given by the constant *k*_3_, whereas *k*_2_ is the cost of resistance. When there is no antibiotic stress, resistance is costlier than producing public goods. When the antibiotic stress is high, producing public goods is costlier than resistance.
— *N* is arbitrarily chosen to be 4, while all the other constants are equal to unity per time unit.

### 2.2. Simulation

The simulations were performed using COPASI v. 4.27 and its deterministic solver LSODA [26]. All the model parameters have been adapted from the paper by Wilhelm & Heinrich [23]. We use hour as the time unit, so that the rate constants have the values 1 h^−1^. We run the time course calculation of the system (2.1*a*–*c*) for 25 simulation hours with 1000 steps each of size 0.025 h (1.5 min). Moreover, we use methods from evolutionary game theory. The game theoretical modelling will be explained in the Results section.

## 3. Results

### 3.1. Results of the ODE model

**Figure 2.**
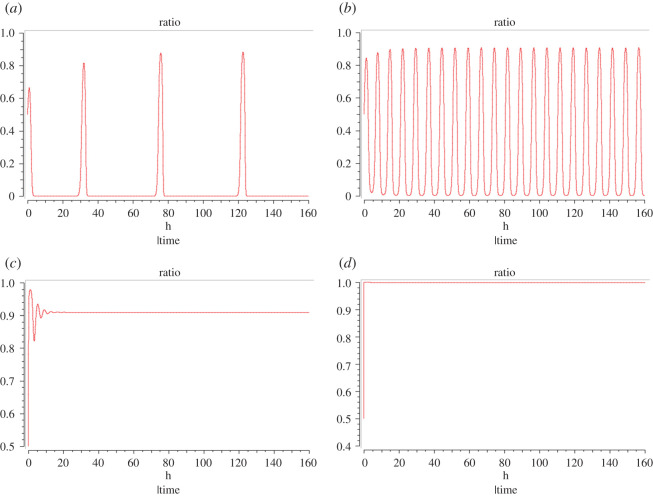
Ratio of *R* to that of the sum of *R* and *P* versus time. (*a*) When *k*_3_ < *k*_2_ (specifically *k*_2_ = 1, *k*_3_ = 0.1 in the simulation shown); (*b*) when *k*_2_ approximately equals *k*_3_ (here *k*_2_ = *k*_3_ = 1); (*c*) when 2*k*_2_ < *k*_3_ (here *k*_2_ = 1, *k*_3_ = 10); (*d*) when *k*_2_ ≪ *k*_3_ (here *k*_2_ = 1, *k*_3_ = 1000). The system transitions from spike-like oscillations to sinusoidal ones, then to damped oscillations, leading to a steady state and finally to a nearly monotonic relaxation to a steady state.

Based on the different antibiotic stresses, we can observe three different behaviours from the subpopulations. Figure 2 shows the ratio of *R* to that of the sum of *R* and *P* over time for three different levels of antibiotic stress. At zero stress, it is observed that the public good production is the dominant strategy (figure 2*a*). However, we observe periodic spikes in the proportion of *R* cells. These spikes can be attributed to bet-hedging cycles where the community invests in making *R* cells in spite of high costs and zero stress. At moderate costs, we observe that the ratio of *R* cells oscillates in sinusoidal form with a shorter period. This implies that there is a constant switching in the dominant subpopulations owing to similar costs of production for either subpopulation (figure 2*b*). As the public goods get costlier, the *R* subpopulation emerges to be the dominant strategy. This state is reached through damped oscillations (figure 2*c*). For very high costs, the steady state is reached by a monotonic relaxation with practically no oscillation (figure 2*d*). In mathematical terms, this corresponds to a stable node. It is unlikely that this case is of biological relevance because public goods are only produced if costs are not too high.

**Figure 3.**
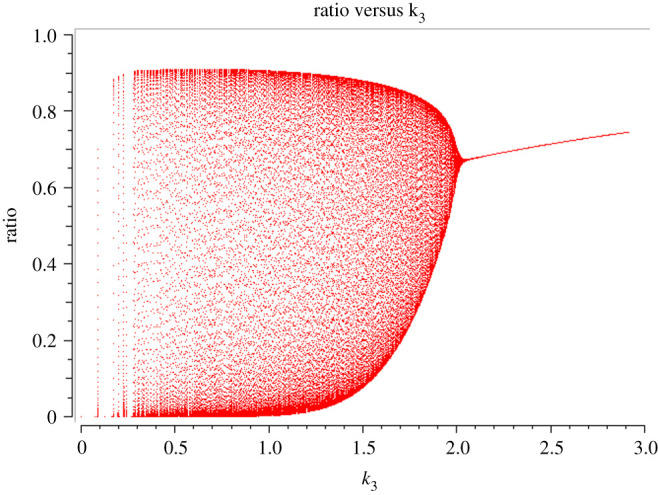
Bifurcation diagram of *R*/(*R* + *P*) against *k*_3_. The ratio oscillates, for example, at *k*_3_ = *k*_2_ (=1), but as *k*_3_ increases, the amplitude of oscillation reduces until a steady state is reached at *k*_3_ = 2. The two arms of the convex hull represent the maxima and minima of oscillation (amplitude).

The bifurcations separating the different dynamic regimes can be determined in the same way as shown in our earlier paper [16]. The effect of changing *k*_3_ for this model is shown in figure 3. This type of transition is called Hopf bifurcation.

### 3.2. Describing the results using evolutionary game theory

#### 3.2.1. Three-strategy two-player game

**Figure 4.**
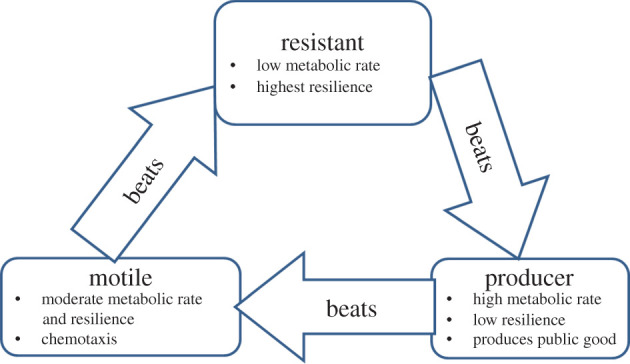
The dynamics of biofilm subpopulations as a three-strategy game. No single strategy is better than either of the remaining strategies.

The observations of this model can be described using evolutionary game theory. For this, we consider a three-strategy game, where the subpopulations are considered as the three strategies. In order to assign payoffs correctly, we must consider the advantages and disadvantages of each of the subpopulation. Subpopulation *R* has the lowest metabolic activity and hence it can withstand extreme conditions such as nutrient limitation and chemical attacks. Subpopulation *P* on the other hand is the most susceptible to chemical stress such as antibiotics due to high metabolic activity. It has been shown that some antimicrobials such as β-lactam antibiotics target metabolically active cells [27]. Subpopulation *M* is not as active metabolically as subpopulation *P*; hence, it is less susceptible to antimicrobials in comparison. But it is motile and is therefore capable of chemotaxis (i.e. *M* cells can move away from antibiotics and closer to nutrients). Thus, no subpopulation has a clear advantage over the other two and the game is comparable to the well-known rock–paper–scissors (RPS) game (figure 4).

The three subpopulations are considered as the three strategies and we can compare the payoffs in the all-against-all fashion in a payoff matrix (table 1). Based on the interaction given in figure 4, we award the ‘winner’ 1 point while the loser loses 1. Draws/ties, such as the ones resulting from playing the same strategies, are given no points. Thus, it is a zero-sum game.

**Table 1.** Payoffs for the three-strategy, two-player game.

player 2 player 1	*R*	*M*	*P*
*R*	0\0	−1\1	1\−1
*M*	1\−1	0\0	−1\1
*P*	−1\1	1\−1	0\0

As seen from table 1, for every strategy of player 1, player 2 can change to a better strategy and vice versa, indicating that there is no Nash equilibrium in pure strategies. A similar scenario has also been described for the RPS game, either by payoff matrices [28] or by an ODE approach [29–31]. The description by ODEs predicts, depending on parameter values, either undamped oscillations by which one type outcompetes the other two, coexistence of the three types in an oscillatory way or stationary coexistence [29].

All the results obtained in evolutionary game theory for the RPS game can be applied to the above system. In particular, there is no pure Nash equilibrium. The only mixed Nash equilibrium is that all the three strategies are played with a probability of 1/3. Other fractions can easily be simulated by taking other payoff values. One way to do this is to simply cycle through the strategies successively. This implies a cyclic dominance of different subpopulations. This explains the life cycle of the community.

#### 3.2.2. Two-strategy two-player game

Another method to model this is by considering a two-strategy game. For this, we exclude the *M* cell population and only consider *R* cells and *P* cells as strategies. We then consider the interaction of a typical single cell with a group of mature cells in the community. The single nascent cell could either be of type *R* or *P*. In evolutionary game theory, this is often called an invading rare mutant (where ‘invading’ does not necessarily mean invasion from the outside, it could also mean occurrence by mutation). Similarly, the group of mature cells within the community can be of type *R* or *P*. The payoffs for this two-strategy game are given in table 2, white columns.

**Table 2.** Payoffs of the two-strategy two-player game. The three cases based on the level of antibiotic stress reflected by the cost of resistance in relation to public goods production are given in blue (no stress, i.e. *k*_3_ < *k*_2_), white (moderate stress, i.e. *k*_3_ = *k*_2_) and red (high stress, i.e. *k*_3_ > *k*_2_) columns, respectively.

player 2	*P* (mature)			*R* (mature)		
player 1	*k_3_ < k_2_*	*k*_3_ = *k*_2_	*k_3_ > k_2_*	*k_3_ < k_2_*	*k*_3_ = *k*_2_	*k_3_ > k_2_*
*P* (nascent)	2\3	1\2	0\1	1.5\1.5	0.5\1.5	−0.5\1.5
*R* (nascent)	2\1	2\0	2\−1	0\2	0\2	0\2

The best outcome for a nascent resistant cell is against the mature producer—it gets access to the public goods without any costs. Hence, it gets a payoff of 2. The mature producer cells, on the other hand, have a payoff of zero, since they have to share their nutrients with the new ‘freeloader’ cell.

A nascent resistant cell is not as resilient when compared with the matured resistant cells in the community and is thus outcompeted. For the mature resistant cells, the newcomer is an added taskforce of resistant cells and thus a much desired outcome. The nascent resistant cell gets a payoff of 0 while the matured resistant cells get a payoff of 2.

The nascent *P* cell interacting with the mature *R* cells is exploited by the *R* cells for its public goods. Thus, it gets a payoff of 0.5, while the resistant cells get a payoff of 1.5. On the other hand, a nascent *P* is added task force for public goods production; hence, the mature *P* cells benefit from this and have a payoff of 2. The nascent *P* cell also gets a payoff of 1.

This describes the game when the antibiotic stress is moderate (i.e. when *k*_3_ = *k*_2_). For the remaining two cases, one can simply add one point to the payoff of all *P* type strategies in order to obtain the blue column, which describes the dynamics of the same game in the absence of the antibiotic stress (i.e. when *k*_3_ < *k*_2_). Similarly, deducting one point from the payoff of all *P* strategies helps us obtain the dynamics of the game when the antibiotic stress is high (i.e. *k*_3_ > *k*_2_), shown in the red column.

It can be seen from the payoff matrix that there is no pure Nash equilibrium when *k*_3_ = *k*_2_ (white columns) and there is no evolutionarily stable strategy. This is because for any change in the strategy of mature cells, the nascent cells can respond with a corresponding change in their strategy as well and one can expect a cyclic nature of the strategies leading to oscillations in the ratio of resistant cells. On the other hand, one can expect public goods production to be a dominant strategy when *k*_3_ < *k*_2_ (blue columns); however, since there is no difference in the payoffs of a nascent resistant cell when compared with a nascent producer when playing against matured producers, an occasional switch to the resistant type may be made by the nascent cell. Furthermore, when *k*_3_ > *k*_2_ (red columns), one can expect an unequivocal domination by resistance cells. This observation is well reflected in the three-variable model mentioned above (figure 2*c*).

## 4. Discussion

This study focuses on the population dynamics in a community of a unicellular social organism. Several efforts have been made with respect to *B. subtilis* biofilms in the same direction before [32–36]. This study employs an ODE-based minimal model in order to describe the population dynamics in a community of a unicellular social organism. The mathematical model has been proposed by Wilhelm & Heinrich [23] to describe chemical reactions and more recently, it has been also used to describe periodic halting in the expansion of a biofilm of *B. subtilis* [16,17].

The model consists of three variables, each representing a subpopulation in the community. Figure 1 describes the relationship of the model variables with each other. As per the assumption, both *R* and *P* type cells are crucial components of the sessile community. The term *k*_2_*RP* represents the degree of bet hedging, where *k*_2_ is the cost of resistance. It also follows that the higher the investment into public goods, the higher is the degree of bet hedging. The interplay between the three subpopulations is illustrated in figure 1.

Based on this relationship, one can visualize a game similar to the RPS game. Table 1 describes the payoffs of this three-strategy game. Such a game with oscillatory dynamics was also described for bacteriocin production in bacteria using a Lotka–Volterra model [29]. There is a body of literature about modelling the RPS game by ODEs [29–31] and even partial differential equations (PDEs) [37,38]. Our model is an even simpler description of the game and thus a minimal model for the RPS game. Indeed, one may use PDEs to consider complex spatial effects like travelling spiral waves of the three types or gradients of the antibiotic. In [37], such travelling spiral waves were found. Another approach would be to use agent-based modelling [39,40]. Here, we want to explore the phenomenon strictly with ODEs. What is more interesting is that our model can be applied to not just bacteria but any communal organism with similarly interacting subpopulations. A further similarity of our approach to that of Neumann & Schuster [29] is that the Hopf bifurcation is obtained for the bacteriocin parameter, similar to the antibiotic stress as discussed here.

In the model presented above, players can change strategies, while in the previous approach by Neumann & Schuster [29], the fraction of strategies changes by different growth rates and competition between the strategies. It is interesting that both cases can be described by the concepts of evolutionary game theory.

In order to gain further insight into the population dynamics, we study the two strategies of resistance and biofilm production further. It can be seen from figure 2*a*, which depicts the time course of the ratio of resistant cells to that of the sum of resistant cells and producers, in the absence of the antibiotic stress (*k*_3_ < *k*_2_), we observe that biofilm production is the dominant strategy. Furthermore, one can also observe periodic ‘resistance spikes’, suggesting that the population undergoes bet-hedging cycles in order to have a reserve of cells that can survive adverse conditions. This warrants experimental validation in order to conclusively state that such bet-hedging cycles occur, although there have been reports on the sporulation cycles [41] and bet hedging [42,43] in bacteria. The study indicates that bacteria undergo sporulation even in the absence of nutrient stress as a bet-hedging strategy. Furthermore, the period of these cycles is also not depicted accurately in this model since the rate constants are chosen arbitrarily because the goal here is to describe the underlying mechanism qualitatively and non-specifically.

This observation can be described using a two-strategy two-player game. In table 2, the blue columns represent this particular case where the antibiotic stress is absent and hence resistance is a costly investment (i.e. *k*_3_ < *k*_2_). Comparing the payoffs, one can expect that the Nash equilibrium would be to produce the public goods. However, since the payoffs for the nascent cell playing either strategy against the matured producer cells are the same, it is also not surprising to see an occasional rise in the resistant type subpopulation.

Figure 2*b* describes the case when the antibiotic stress is moderate, and hence investing resources in resistance is favourable and, in this case, *k*_3_ = *k*_2_, meaning that producing public goods costs as much as resistance. One can observe a cycling of strategies between the resistant and the producer types. This is further explained by the two-strategy game. The white columns in table 2 depict this case and one can see that there is no pure Nash equilibrium. This means that for every strategy chosen by player 1, player 2 can improve its payoff by switching its own strategy. We can expect that the producer strategy is a suboptimal strategy where both players have relatively high payoffs. We may also hypothesize that when the community is sufficiently large, this cyclic switching of strategies might switch to a stable steady state where the ratio remains constant. This has been observed in the modified version of this ODE system by Garde *et al.* [17].

Finally, figure 2*c* describes the case when the antibiotic stress is the highest and investing in public goods instead of resistance is costlier, as such, *k*_3_ > *k*_2_. As expected, the resistant cells emerge as the dominant subpopulation. This can be thought of as extreme resource limitation where the public goods become extremely costly to produce. In such a scenario, it is ideal for the community to produce resistant cells in order to ensure that it can survive this starvation phase until the nutrients are available once again. This scenario is also described in table 2 in the red column. One can conclude that in this case, resistance is undoubtedly the dominant strategy. Cyclic behaviour is possible in symmetric three-strategy game, while a two-strategy game needs to be asymmetric to yield cycles.

This model can be modified in order to describe a particular bacterial biofilm. In such a scenario, the resistant cells can be thought of as spores and the public goods can be thought of as the biofilm matrix. In this study, however, we only aim to put forth a generalized mechanism of the (non-cognitive) decision-making happening in the population of a unicellular communal organism (e.g. by entering the next developmental stage caused by an epigenetic switch). The ODE-based system also serves as a minimal model for the three-strategy RPS game with cyclic dominance of strategies. Further, the two-strategy game suggests that, as the antibiotic stress increases, the optimal strategy shifts from producing public goods towards producing resistance. Moreover, it also suggests that even in the absence of the antibiotic stress, the community invests in resistance as a bet-hedging strategy.

## Data accessibility

No custom code and datasets were used within this study.
